# Molecular assessment of the phylogeny and biogeography of a recently diversified endemic group of South American canids (Mammalia: Carnivora: Canidae)

**DOI:** 10.1590/1678-4685-GMB-2015-0189

**Published:** 2016-07-25

**Authors:** Ligia Tchaicka, Thales Renato Ochotorena de Freitas, Alex Bager, Stela Luengos Vidal, Mauro Lucherini, Agustín Iriarte, Andres Novaro, Eli Geffen, Fabricio Silva Garcez, Warren E. Johnson, Robert K. Wayne, Eduardo Eizirik

**Affiliations:** 1 Departamento de Química e Biologia, Centro de Educação, Ciências Exatas e Naturais (CECEN), Universidade Estadual do Maranhão (UEMA), São Luís, MA, Brazil; 2 Departamento de Genética, Instituto de Biociências, Universidade Federal do Rio Grande do Sul (UFRGS), Porto Alegre, RS, Brazil; 3 Departamento de Biologia, Universidade Federal de Lavras (UFLA), Lavras, MG, Brazil; 4 Departamento de Biología, Bioquímica y Farmacia, Universidad Nacional del Sur, Bahía Blanca, Argentina; 5 Center for Advanced Studies in Ecology & Biodiversity (CASEB), Pontificia Universidad Catolica & Fundacion Biodiversitas, Santiago, Chile; 6 Consejo Nacional de Investigaciones Científicas y Técnicas, and Wildlife Conservation Society-Argentina, Junín de los Andes, Neuquén, Argentina; 7 Department of Zoology, Tel Aviv University, Israel; 8 Laboratório de Biologia Genômica e Molecular, Faculdade de Biociências, Pontifícia Universidade Católica do Rio Grande do Sul (PUCRS), Porto Alegre, RS, Brazil; 9 Smithsonian Conservation Biology Institute, Washington, DC, USA; 10 Department of Ecology and Evolutionary Biology, University of California, Los Angeles, CA, USA; 11 Instituto Pró-Carnívoros, Atibaia, SP, Brazil

**Keywords:** Mitochondrial DNA control region, Lycalopex, Canidae, Carnivora

## Abstract

To investigate the evolution and biogeography of an endemic group of South American foxes, we examined mitochondrial DNA control region sequences for 118 individuals belonging to all six extant species of the genus *Lycalopex*. Phylogenetic and molecular dating analyses supported the inference that this genus has undergone a very recent and rapid radiation, stemming from a common ancestor that lived *ca.* 1 million years ago. The Brazilian endemic *L. vetulus* was supported as the most basal species in this genus, whereas the most internal group is comprised by the recently diverged (*ca.* 350,000 years ago) Andean/Patagonian species *L. griseus* and *L. culpaeus*. We discuss the inferred phylogenetic relationships and divergence times in the context of the current geographic distributions of these species, and the likely effects of Pleistocene climatic changes on the biogeography of this group. Furthermore, a remarkable finding was the identification of multiple individuals classified as *L. gymnocercus* bearing mtDNA haplotypes clearly belonging to *L. griseus*, sampled in regions where the latter is not known to occur. At a minimum, this result implies the need to clarify the present-day geographic distribution of each of these fox species, while it may also indicate an ongoing hybridization process between them. Future testing of this hypothesis with in-depth analyses of these populations is thus a priority for understanding the history, evolutionary dynamics and present-day composition of this endemic Neotropical genus.

## Introduction

The first representatives of the family Canidae entered South America in the late Pliocene and early Pleistocene, coming from North America through the Panama Isthmus (formed approximately 3 million years ago [mya]), and then radiated to achieve their present diversity (Berta, 1987). Currently there are ten canid species endemic to South America, representing the largest diversity of this family on any continent. This diversity has been attributed to their generalist and opportunistic feeding strategies that utilize vertebrate prey as well as fruits and invertebrates, and their adaptation to a wide variety of habitats (Berta, 1987; Ginsberg and MacDonald, 1990; Wozencraft, 2005).

Of the ten living species of South American canids, eight are often referred to as foxes, and recognized as a monophyletic assemblage comprising the genera *Cerdocyon, Lycalopex*, and *Atelocynus* (Wayne *et al.*, 1997; Zrzavy and Ricankova, 2004; Slater *et al.*, 2009; Perini *et al.*, 2010; Prevosti, 2009). These species have similar karyotypes, suggesting a recent divergence (2n=74; NF=76 - *A. microtis, L. gymnocercus, L. griseus, L. culpaeus, L. sechurae, L. gymnocercus and L. vetulus*; 2n = 74; NF=106 - *Cerdocyon thous* [Brum-Zorrila and Langguth, 1980; Wayne *et al.*, 1987; Wayne, 1993]). Although several previous studies have addressed the evolutionary relationships among these foxes using morphological and/or molecular data (*e.g.* Wayne *et al.*, 1997; Lyras and Van Der Geer, 2003; Zrzavy and Ricancova, 2004; Bardeleben *et al.*, 2005; Lindblad-Toh *et al.*, 2005; Prevosti, 2009; Slater *et al.*, 2009; Perini *et al.*, 2010), the resolution of their phylogeny remains elusive, especially with regard to the species belonging to the genus *Lycalopex* (including *Pseudalopex* – see below). These canids will be treated here as *Lycalopex vetulus* (hoary fox), *L. gymnocercus* (pampas fox), *L. culpaeus* (culpeo), *L. fulvipes* (Darwin's fox), *L. griseus* (chilla) and *L. sechurae* (Sechuran fox), following Wozencraft (2005).

**Figure 1:**
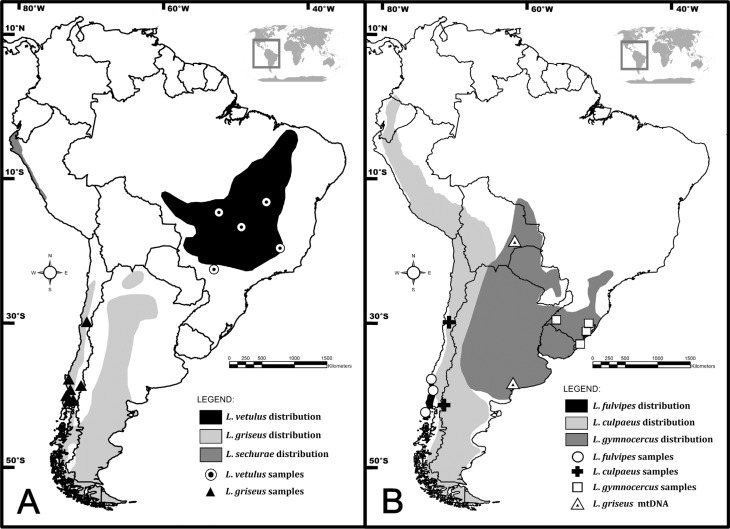
Maps showing the current geographic distribution of *Lycalopex* species (modified from Courtenay and Maffei (2004) and approximate sample collection sites. Triangles in panel B indicate sampling localities of individuals initially labeled as *L. gymnocercus*, but whose mtDNA haplotypes group within the *L. griseus* clade. Individuals with unknown geographic origin were not included in the map (see Table S1 for more details).

Furthermore, the precise geographic range of these species is still not known in full detail, although some broad distributional patterns are well documented (Figure 1). *L. culpaeus* is distributed along the Andes and hilly regions of western South America, from southern Colombia to Tierra del Fuego. *L. fulvipes* is endemic to costal Chile. *L. griseus* is widespread in areas of plains and mountains on both sides of the Andes, from northern Chile south to the Strait of Magellan (introduced by humans into the island of Tierra del Fuego in 1953). *L. gymnocercus* is currently thought to range from eastern Bolivia and western Paraguay to central Argentina and southern Brazil. *L. sechurae* occurs on the Pacific coast of Peru and Ecuador. Finally, *L. vetulus* is endemic to the Cerrado biome and adjacent areas in central Brazil (Sillero-Zubiri *et al.*, 2004).

Several taxonomic schemes for these species have been suggested based on different methods. Langguth (1975), based on ecological and morphological data, suggested two taxa: (i) genus *Lycalopex* Burmeister, 1854 and (ii) *Pseudalopex* Burmeister, 1856 as a subgenus of *Canis*. The former contained only *Lycalopex vetulus*, the type species for this genus, while the latter contained *Canis* (*Pseudalopex*) *culpaeus*, *C*. (*P*.) *gymnocercus*, *C*. (*P*.) *griseus* and *C*. (*P*.) *sechurae*. Subsequently, Clutton-Brock *et al.* (1976), using morphological and behavioral data, included all these species in the genus *Dusicyon* C. E. H. Smith, 1839, originally proposed for the now extinct Falkland Island "wolf", *D*. *australis* (Wozencraft, 2005). Berta (1987), based on the fossil record and cladistic analyses of morphological data, proposed that the genus *Pseudalopex* should include *P. griseus, P. gymnocercus, P. culpaeus, P. vetulus*, *P. sechurae* and the extinct species *P. peruanus*. Subsequently, Zunino *et al.* (1995) grouped *P. gymnocercus* and *P. griseus* into a single species, *Lycalopex gymnocercus*, supporting the use of *Lycalopex* as the generic name for *L. culpaeus, L. vetulus* and *L. sechurae* (*L. fulvipes* was also considered to be a synonym of *L. gymnocercus* in that study).

Additional classifications of this group have been suggested (Thomas, 1914; Kraglievich, 1930; Cabrera, 1931; Osgood, 1934; Hough, 1948; Thenius, 1954; Van Gelder, 1978), illustrating the ongoing taxonomic instability in this Neotropical clade throughout the 20^th^ century. Ultimately, this confusion is a reflection of the underlying uncertainty regarding the species limits and phylogenetic relationships among these foxes, highlighting the need for additional work focusing on this group. Recent analyses (*e.g.* Prevosti, 2009; Slater *et al.*, 2009; Perini *et al.*, 2010; Prevosti *et al.*, 2013) have contributed to this debate by exploring larger data sets composed of molecular and/or morphological characters, but still have not conclusively settled these relationships, illustrating the difficulty in achieving a robust phylogeny for this group.

Mitochondrial DNA (mtDNA) segments are useful in evolutionary studies of recent divergence processes in animals, due to their relatively high substitution rate, maternal inheritance, and absence of recombination (Schlötterer, 2004). In spite of limitations derived from these same features, mtDNA segments remain an important source of information in the case of population studies, phylogeography and phylogenetic studies of closely related species, since these rapidly evolving sequences with lower effective population size are often quite informative in attempts to capture recent episodes of taxon divergence. In particular, the fast-evolving mtDNA control region (CR) may be best suited to reconstruct very recent divergence processes involving intra-specific lineages or closely related species, such as the *Lycalopex* group (whose overall phylogeny has so far not been studied with the CR). Therefore, in this study we employed mtDNA CR sequences to investigate the evolutionary history of *Lycalopex* foxes and their recent radiation in South America.

## Material and Methods

### Biological samples

We collected biological material from 117 Neotropical canids of the genus *Lycalopex* (Figure 1 and Table S1), including 32 *L. culpaeus*, 24 *L. gymnocercus*, 27 *L. vetulus*, 6 *L. fulvipes*, and 28 *L. griseus* (six of which had been initially identified as *L. gymnocercus*; see Figure 1 and Discussion). Five *Cerdocyon thous* individuals (which had been previously sequenced by Tchaicka *et al.*, 2007) were included as outgroups.

Blood samples (preserved in a saturated salt solution of 100 mM Tris, 100 mM EDTA and 2% SDS) were collected from captive individuals, as well as wild animals captured for field ecology studies. Tissue samples were obtained from road-killed individuals and preserved in 95% ethanol.

### DNA extraction and amplification

Genomic DNA was extracted from samples using a standard phenol/chloroform protocol (Sambrook *et al.*, 1989). The 5' portion of the mtDNA control region, containing the first hypervariable segment (HVS-I), was amplified by the polymerase chain reaction (PCR; Saiki *et al.*, 1985) using the primers MTLPRO2 and CCR-DR1 (Tchaicka *et al.*, 2007), or H16498 (Ward *et al.*, 1991) as an alternative reverse primer. PCR mixtures consisted of 2 μl of 10X buffer, 1.5 mM MgCl_2_, 0.2 mM of dNTPs, 0.2 μM of each primer, 0.75 unit *Taq* polymerase (Invitrogen) and 1-3 μl of empirically diluted template DNA. Thermocycling conditions included 10 initial cycles of "touchdown", with 45 s denaturing at 94 °C, 45 s annealing at 60-51 °C, and 90 s extension at 72 °C. This was followed by 30 cycles of 45 s denaturing at 94 °C, 30 s annealing at 50 °C and 90 s extension at 72 °C. Products were examined on a 1% agarose gel stained with ethidium bromide, purified using shrimp alkaline phosphatase and exonuclease I, sequenced with ABI chemistry and analyzed with an ABI-PRISM 3100 automated sequencer. Sequences generated for this study are deposited in GenBank (accession numbers JX890309 - JX890389). In addition to these sequences, one previously published partial sequence of the mtDNA control region of *Lycalopex sechurae* (Yahnke *et al.*, 1996) was included in the analyses, so that a total of 118 individuals (representing all known species of this genus) was analyzed.

### Sequence, phylogenetic relationships and population genetics analysis

Sequences were verified and corrected using Chromas (Technelysium) or Sequencher (Gene Codes Inc.), aligned using the ClustalW algorithm implemented in MEGA 6.0 (Tamura *et al.*, 2013) and visually checked. Sites or segments that could not be unambiguously aligned were excluded from all analyses. Initial sequence comparisons and assessments of variability, such as computing the number of variable sites and nucleotide diversity (π per nucleotide site, the probability that two randomly chosen homologous nucleotides are different in the sample) were performed in MEGA 6.0 using Kimura 2-parameter distances and 1000 bootstrap replicates. Estimates of gene diversity (h, the probability that two randomly chosen mtDNA lineages were different in the sample) were computed in Arlequin 3.11 (Excoffier *et al.*, 2005) with 10,000 permutations to assess their variance.

We reconstructed the phylogenetic relationships among *Lycalopex* haplotypes using the Bayesian approach implemented in Beast 1.8.0 (Drummond *et al.*, 2012). We included only complete sequences (*i.e.* containing no missing data), so as to maximize the stability and reliability of the inferred phylogeny. We estimated the best-fit molecular model of evolution for this data set with Modeltest 3.6 (Posada and Crandall, 1998), using the Akaike Information Criterion. The selected model (GTR+G+I) was then incorporated in the analysis. We ran the Markov Chain Monte Carlo (MCMC) process in Beast for 100 million generations, with the data sampled every 1,000 steps, and discarding the initial 10% as burn-in.

In addition to the phylogenetic analysis, we also investigated the relationships among *Lycalopex* haplotypes using a median-joining network approach, which was performed with Network 4.6.1.2 (Fluxus Technology). Since this method allows for ancestor-descendant relationships among haplotypes, as well as displays genealogical ambiguities more clearly than a tree-based approach, it is expected to be useful in the analysis of this recently diversified group. Moreover, since our data set included multiple individuals per species, we used this approach to assess species-level monophyly of mtDNA lineages, as well as instances of apparent ‘swaps' indicative of erroneous identification or inter-species hybridization (see Results).

To estimate divergence times within this genus, we used two methods. In the first one, we performed a Bayesian estimation using Beast, assuming an uncorrelated lognormal relaxed molecular clock. This analysis was calibrated with the mean substitution rate (μ = 3.68x10^-8^/year) estimated for the same CR segment in canids by Tchaicka *et al.* (2007), based on available data from grey wolf and coyote. In the second method, we employed a population-genetic approach based on the equation d_xy_=2μT (Nei, 1987), using the estimated mtDNA divergence (d_xy_ as implemented in Mega, with K2P distances [a simpler model was incorporated here, relative to the Beast analyses, to minimize the variance around parameter estimates]) between species or groups of species, and the same substitution rate mentioned above. The divergence time between mtDNA lineages was calculated considering the 95% confidence interval (CI = ± 2SE) for all values of d_xy_. Using this interval for the calibration node, we obtained low, medium and fast substitution rate estimations (2.02x10^-8^, 3.68x10^-8^ and 5.34x10^-8^/year, respectively), which were then applied to the equivalent d_xy_ interval estimated for each node. This approach allowed a conservative estimate of uncertainty in the dating of these rapid divergences, while providing a robust assessment of their overall temporal framework.

## Results

**Figure 2:**
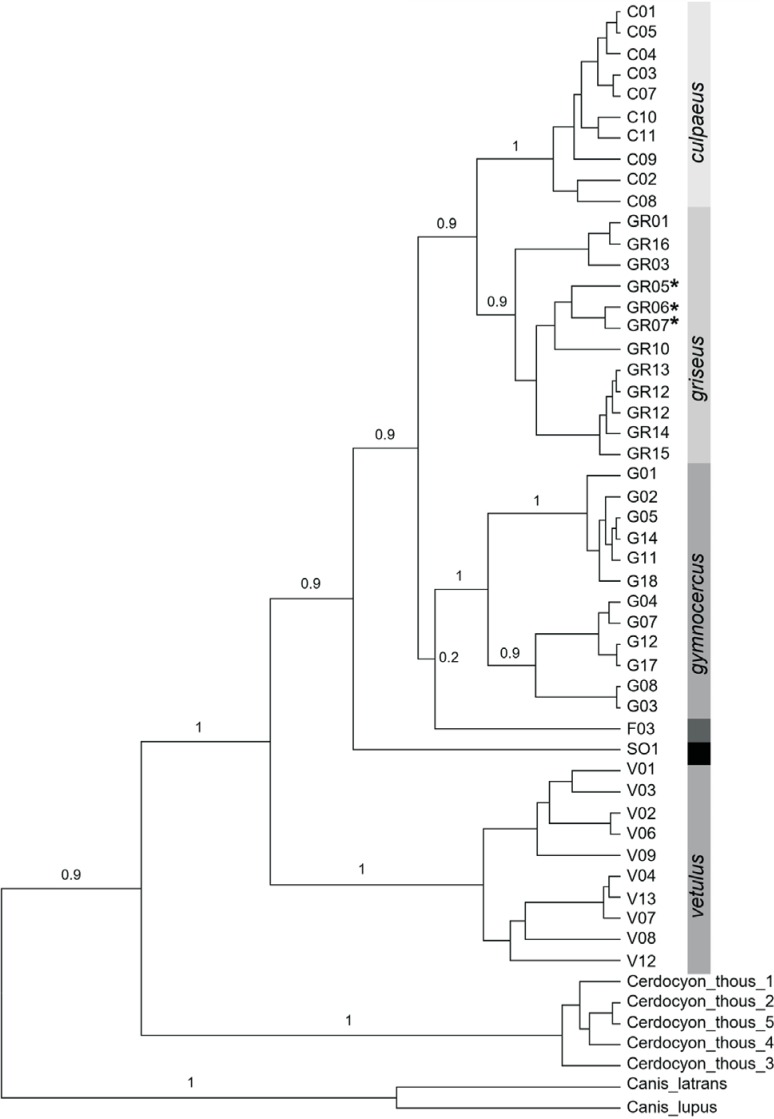
Bayesian phylogeny (built with the GTR+G+I model) of 46 *Lycalopex* spp. haplotypes based on 588 bp of the mitochondrial DNA control region (only complete sequences were included in the analysis). Five *Cerdocyon thous* haplotypes were used as outgroups (see Figure S1 for results with additional outgroups). Labels are haplotype identification numbers (as coded in the Table S1: C – *L. culpaeus*; GR – *L. griseus*; G – *L. gymnocercus*; F – *L. fulvipes*; S - *L. sechurae*; V – *L. vetulus*). Values above branches indicate the Bayesian posterior probability for the adjacent node (support values are shown only for the main clades retrieved in the phylogeny). Asterisks indicate haplotypes deriving from samples initially labeled as *L. gymnocercus*, but whose phylogenetic position was nested within the *L. griseus* clade.

Sixty-nine different haplotypes were identified with the 588-base pair (bp) segment sequenced for *Lycalopex* species, defined by 220 variable sites and 193 parsimony-informative sites. Base composition was biased, with a deficit of guanine (T=31.1%; C=24.6%; A=26.6%; G=17.6%). No haplotypes were found to be shared among species. The estimated diversity indices are shown in Table 1. The phylogenetic analysis generated a tree topology in which several nodes were supported with high posterior probability (Figure 2). The main features of the reconstructed tree were: (i) *L. vetulus* as the most basal species in the genus *Lycalopex*; (ii) high support (PP > 0.9) for the monophyly of every species for which we had more than one individual (*L. vetulus*, *L. gymnocercus*, *L. culpaeus* and *L. griseus* [but see below for a special case within *L. griseus*]); and (iii) a well-supported, sister-group relationship between *L. culpaeus* and *L. griseus*. The single *L. fulvipes* sequence was placed as a sister-group to *L. gymnocercus* (albeit with low support), and both were included in a broader group that also contained *L. culpaeus* + *L. griseus*. This clade was in turn the sister-group of the single *L. sechurae* sequence (see Figure 2).

**Table 1:** Diversity indices (gene [h] and nucleotide [π] diversity) observed in *Lycalopex* species control region sequences.

Species	N	h	Π	Number of haplotypes	Number of variable sites	Number of parsimony informative sites
*L. culpaeus*	32	0.8004 +/- 0.0407	0.005 ± 0.002	11	16	10
*L. vetulus*	27	0.9323 +/- 0.0352	0.023 ± 0.004	16	51	33
*L. griseus*	28	0.9398 +/- 0.0311	0.023 ± 0.005	17	50	27
*L. gymnocercus*	24	0.9723 +/- 0.0209	0.022 ± 0.004	17	44	29
*L. fulvipes*	6	0.6000 +/- 0.2152	0.009 ± 0.004	3	5	4

**Figure 3:**
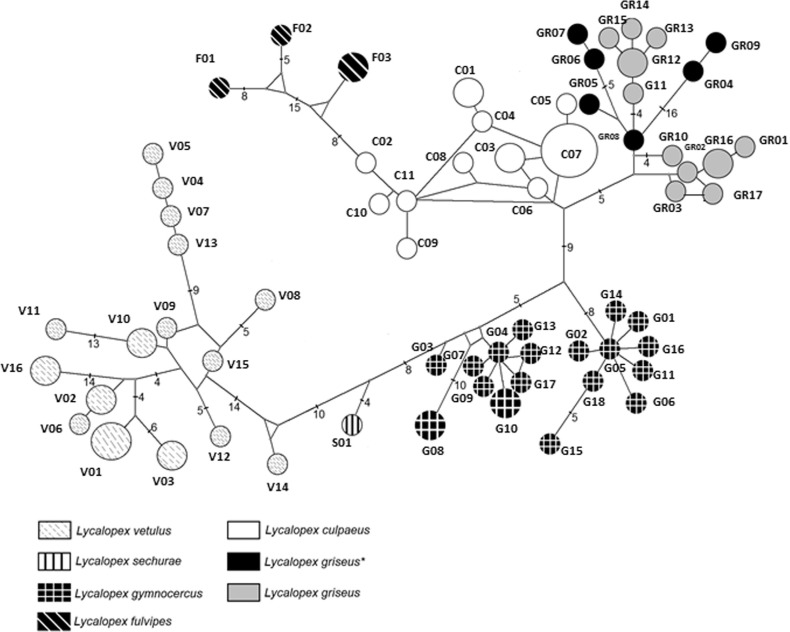
Median-joining network of *Lycalopex* mtDNA control region haplotypes. Each circle represents a distinct haplotype (circle area is proportional to the haplotype's global frequency in the sample), color-coded per species as indicated in the internal legend. Numbers located on connecting branches represent the number of substitutions inferred to exist between haplotypes (branches with no number imply a single substitution). Haplotypes shown in black (and marked with an asterisk in the legend) were sampled in six individuals that were initially identified as *L. gymnocercus* (see text and Table S1 for details).

The haplotype network (Figure 3) also revealed interesting patterns, which were broadly consistent with the phylogenetic tree. An interesting difference was the position of *L. fulvipes*, which was inferred here to be nested within the diversity of *L. culpaeus* haplotypes. There was evidence of population expansion (*i.e.* a star-like network, with few mutations between the haplotypes) within species-level clusters. On the other hand, only in the *L. gymnocercus* group did we observe sub-groups that showed some evidence of geographic structure (see Figures 2 and 3).

Interestingly, both the phylogenetic analysis and the haplotype network indicated that, while the *L. gymnocercus* sequences from southern Brazil did form a monophyletic group, mtDNA lineages from six individuals collected in Bolivia and Argentina, and identified as *L. gymnocercus* in the field (Figure 1, Table S1), were strongly placed as members of the *L. griseus* clade (Figures 2 and 3).

The two methods used to estimate the divergence time between clades yielded broadly similar results. We conservatively estimated that *Cerdocyon* and *Lycalopex* diverged between 1–3 mya. *L. vetulus* seems to have diverged from other *Lycalopex* species *ca.* 1 mya, while the youngest event, the divergence between *L. culpaeus* and *L. griseus*, is inferred to have occurred very recently, *ca.* 600,000 – 350,000 years ago (ya) (Table 2).

**Table 2:** Divergence time estimated for *Lycalopex* groups with a Bayesian dating analysis and a distance-based approach (see text for details).

Bayesian Inference		
Clade (basal divergence)	Age (ybp)	95% HPD interval
*Cerdocyon* + *Lycalopex*	1,951,000	1,173,000 – 2,767,000
*Lycalopex*	1,353,000	857,000 – 1,889,000
*L. sechurae* + *fulvipes* + *griseus* + *culpaeus* + *gymnocercus*	1,075,000	652,000 – 1,546,000
*L. fulvipes* + *griseus* + *culpaeus* + *gymnocercus*	806,000	531,000 – 1,131,000
*L. fulvipes* + *gymnocercus*	708,000	475,000 – 1,076,000
*L. culpaeus* + *griseus*	607,000	354,000 – 900,000
*L. vetulus*	554,000	325,000 – 830,000
*L. gymnocercus*	505,000	303,000 – 750,000
*L. griseus*	438,000	246,000 – 667,000
*L. culpaeus*	292,000	139,000 – 487,000
Distance-based Inference		
Pair of Clades	Age (ybp)	Lower and Upper bound
*Cerdocyon* X *Lycalopex*	1,125,000	580,000 – 2,821,000
*L. vetulus* X other *Lycalopex*	1,086,000	543,000 – 2,524,000
*L. sechurae* X (*L. fulvipes + griseus + culpaeus + gymnocercus*)	964,000	552,000 – 2,301,000
(*L. fulvipes* + *gymnocercus*) X (*L. griseus + culpaeus*)	896,000	337,000 – 2,376,000
*L. fulvipes* X *gymnocercus*	706,000	243,000 – 930,000
*L. culpaeus* X *griseus*	366,000	140,000 – 965,000

## Discussion

The phylogenetic analysis resolved with confidence several nodes within this group of canids, indicating that *L. griseus* and *L. culpaeus* are sister taxa (Figures 2 and 3) that diverged recently, *ca.* 600,000 – 350,000 ya (Table 2). These results are consistent with a previous molecular study (Yahnke *et al.*, 1996), which reported *ca.* 250,000 – 500,000 ya as the age of this event. In that study, based on a shorter segment of the mtDNA CR (344 bp), *L. griseus* and *L. culpaeus* were not retrieved as reciprocally monophyletic groups, possibly deriving from such a recent divergence that they had not yet achieved complete lineage sorting even for the mtDNA. Our analysis, based on longer sequences, indicates that the two species are well supported as reciprocally monophyletic mtDNA phylogroups.

The low gene diversity, the closely related haplotypes and the absence of substructure in the network analysis support the young origin of *L. culpaeus* in our sampled area, where Cabrera (1931) considered the occurrence of two subspecies: *L. c. culpaeus* and *L. c. magellanicus*. Our results agree with Yahnke *et al.* (1996) and Guzman *et al.* (2009) in recognizing a single genetic and morphological group for this species in central-southern Chile.

The results we obtained for *L. griseus* prompted a more careful comparison with the *L. gymnocercus* data, since some individuals phenotypically identified as *L. gymnocercus* (sampled in Bolivia and Argentina, see Figure 1) were strongly grouped in the *L. griseus* clade. There are no reliable reports of *L. griseus* occurring in these regions, and information on the precise distribution limits of both species is still scarce. Furthermore, although the presently assumed range of *L. gymnocercus* overlaps with that of *L. griseus* in several areas, the presence of sympatric populations has never been reported (Lucherini and Luengos Vidal, 2008). One plausible explanation for our results is that the areas where our samples were collected may be in fact inhabited by *L. griseus* instead of *L. gymnocercus*. An alternative (non-exclusive) hypothesis is that the presence of *L. griseus* haplotypes in these regions may be due to hybridization and mtDNA introgression affecting these populations. Each of these hypotheses will be discussed in detail below.

Since pelage color patterns are very similar between these foxes (Zunino *et al.*, 1995), identifying them can be challenging, which suggests that recording errors may have confused historical reports on these species' natural history and the delimitation of their ranges. Studying foxes of the same region, from Bolivia and Paraguay to central Argentina, Zunino *et al.* (1995) and Prevosti *et al.* (2013) analyzed pelage characters and cranial measurements of *L. griseus* and *L. gymnocercus*. These authors observed a clinal variation in size and color, and concluded that these foxes are conspecific (thus calling them *L. gymnocercus*, the senior name). In contrast, our mtDNA data do not support the merging of these two species into a single unit, since they are not sister-groups in the phylogeny, and seem to represent clearly differentiated evolutionary lineages, at least with respect to their matrilineal history. We thus consider it premature to unite them, and recommend additional taxonomic studies employing an expanded suite of approaches. In particular, it would be important to collect both morphological and molecular data from the same voucher specimens, representing the full geographic range of these species, so that results from the two types of data could be adequately compared.

The hypothesis that secondary hybridization and mtDNA introgression best explains our results should be considered in this context, as it could also account for the clinal pattern of morphological variation observed in some geographic regions (Zunino *et al.*, 1995; Prevosti *et al.*, 2013). The samples analyzed here that were initially labeled as *L. gymnocercus* and that bear *L. griseus* mtDNA might be hybrids between male pampas foxes and female chillas, or further descendants from such a cross. Although inter-species hybridization has so far not been reported for South American foxes, this process has been clearly documented for other canid groups whose members are genetically similar due to recent divergence (*e.g.* Gottelli *et al.*, 1994; Roy *et al.*, 1994; VonHoldt *et al.*, 2011; Wilson *et al.*, 2012). It is therefore plausible to postulate that secondary admixture may also occur in this recently diversified canid genus, a hypothesis that should be investigated in more detail with expanded sampling in these areas and the use of additional molecular markers.

The exact position of *L. fulvipes* within the internal *Lycalopex* clades was not completely consistent among our analyses, but the network indicates a well-defined cluster of haplotypes. This fox lives in costal temperate rainforests of Southern Chile, where it inhabits Chiloe Island and also occurs in sympatry with the chilla and culpeo in small continental areas (Medel *et al.*, 1990; Vilà *et al.*, 2004; D'Elia *et al.*, 2013). Initially, it was described as an endemic insular canid and considered a subspecies of continental *L. griseus* (Redford and Eisenberg, 1992; Wilson and Reeder, 1993; Nowak, 1999). A molecular genetic analysis conducted by Yahnke *et al.* (1996) revealed that Darwin's fox is a distinct species, forming a monophyletic mtDNA lineage that was a sister taxon to the (*L. griseus + L. culpaeus*) cluster, from which it would have diverged in the Pleistocene, *ca.* 275,000 to 667,000 ya. These conclusions are corroborated by the present study, as we estimate a divergence time of *ca.* 700,000 to 800,000 ya between Darwin's fox and its immediately related clades.

The haplotypes of the endemic Brazilian hoary fox *L. vetulus* formed a well-differentiated group, supporting an early divergence of this lineage. Our phylogenetic results strongly supported a basal position for this species within the genus. The estimated time of divergence from the other species was *ca.* 1 – 1.3 mya. Similar values (1.95 mya and 1.3 mya) were obtained for the base of *Lycalopex* by Slater *et al.* (2009) and Perini *et al.* (2010), respectively, using mtDNA (coding regions) and nuclear data. The basal position of *L. vetulus* is in agreement with a phylogeny based on multiple nuclear segments (Lindblad-Toh *et al.*, 2005), although a subsequent study (Perini *et al.*, 2010) reporting the joint analysis of a large supermatrix of molecular and morphological characters retrieved a different resolution (with *L. sechurae* as the most basal species). The full resolution of this portion of the *Lycalopex* phylogeny will benefit from additional analyses that integrate large data sets and compare topologies derived from different types of sources.

Debate about the proper usage of *Lycalopex* or *Pseudalopex* for this group has been ongoing for many years (*e.g.* Cabrera, 1931; Osgood, 1934; Langguth, 1975; Berta, 1987, 1988; Tedford *et al.*, 1995). If the topological resolution achieved here is affirmed by future studies and consolidated for the genus, the basal phylogenetic position of the hoary fox implies that it could be kept in its own genus (*Lycalopex*), while the other species could move back to *Pseudalopex*. Alternatively, the whole cluster could be considered a single genus (*Lycalopex*), as in Wozencraft (2005). Both schemes are compatible with the phylogeny we report here, and this decision will thus be arbitrary. We recommend that this decision be based on criteria such as clade age, morphology, and present usage, which should be established in a broader comparison across all lineages of the family Canidae.

### Inferences on the history of South American foxes

Three different canid invasions from North to South America in the Pliocene or Early Pleistocene have been proposed on the basis of previous inferences of the phylogenetic relationships among extant species. One of them would include the ancestor of the fox group that includes *Lycalopex, Cerdocyon, Atelocynus and Dusicyon* (Langguth, 1975; Wang *et al.*, 2004). Genetic divergence values reported by Wayne *et al.* (1997) and Slater *et al.* (2009) suggest that this divergence occurred before the opening of the Panama land bridge, requiring more than one invasion event.

Contrary to this view, our mtDNA control region data indicate that the divergence between *Cerdocyon* and *Lycalopex* took place *ca.* 1 – 3 mya, suggesting that this episode of speciation has likely occurred in South America, after immigration of a single ancestor through the Isthmus of Panama. A similar result has also been reported by Perini *et al.* (2010), who analyzed morphological and molecular (coding genes) characters. In fact, the oldest fossils assigned to *Lycalopex* (*L. gymnocercus*) are reported from Argentinean deposits of the Uquian age (2.5 to 1.5 mya), while those of *Cerdocyon thous* are recorded only from the Lujanian (800,000 – 10,000 ya). Interestingly, there are North American canid fossils reported from the Miocene/Early Pliocene boundary (6 – 3 mya; Berta, 1987) that have been assigned to the genus *Cerdocyon*, which would challenge this hypothesis. However, these specimens are very fragmentary, and Prevosti (2009), based on osteological analyses, proposed that North American fossils that were previously assigned to *Cerdocyon* are, in fact, related to *Urocyon*. The present dating results, which are congruent with our previous analyses (Tchaicka *et al.*, 2007), support this view that the identification and phylogenetic affinities of these fossils should be reassessed.

It is generally inferred from the fossil record (Langguth, 1975; Berta, 1987) and molecular data (Wayne *et al.*, 1997; Perini *et al.*, 2010) that, subsequently to divergence from *Cerdocyon*, the diversification of *Lycalopex* occurred in South America in the Pleistocene. Our results agree with a Pleistocene radiation of *Lycalopex* (Table 2), indicating that: (i) the oldest extant lineages gave rise to *L. vetulus*, and subsequently to *L. sechurae*, in the Early-Middle Pleistocene; (ii) this was followed by the rapid diversification of the *griseus-culpaeus-gymnocercus-fulvipes* clade, in the Middle Pleistocene; and (iii) finally, by the *griseus-culpaeus* recent split in the Middle-Late Pleistocene.

Extensive environmental changes took place in the Neotropical region during the Pleistocene, which may have influenced this canid radiation. Climatic changes affected the vegetation domains as well as the sea level, producing potential geographic barriers to dispersal or confining species to habitat refuges (Withmore and Prance, 1987; Marroig and Cerqueira, 1997; Eisenberg and Redford, 1999). Canids that had crossed the Panamanian Bridge and possibly dispersed through Andean savanna corridors had expanded their range to Patagonian and Brazilian areas by the Early Pleistocene (Langguth, 1975). At this time, the La Plata-Paraguay depression suffered a marine invasion, potentially connected to the Amazon Basin, possibly isolating a large region of Brazil (Marroig and Cerqueira, 1997). This may account for the isolation of this precursor population into two groups, one located east and the other west of this barrier. The eastern (Brazilian) population would give rise to *L. vetulus*, while the western one would originate the remaining lineages.

During the Pleistocene glacial phases, arid climates dominated some of the equatorial areas and savanna corridors were broken. Some mammal species that became restricted to tropical regions may have become savanna-adapted, and now occur in areas consisting of Cerrado habitat (Whitmore and Prance, 1987; Eisenberg and Redford, 1999). This may be the case of the Hoary fox: its small carnassials, wide crushing molars and the exceptionally large auditory bulla (Clutton-Brock *et al.*, 1976) suggest adaptations to a predominantly insectivorous diet. Their preference for insects now allows them to partition food resources and to coexist with other sympatric canids such as the maned wolf (*Chrysocyon brachyurus*) and the crab-eating fox (*Cerdocyon thous*) (Juarez and Marinho-Filho, 2002).

The center of the *Lycalopex* radiation has been proposed by Berta (1987) to have been in central Argentina, whereas Langguth (1975) suggested that central Brazil was the most likely region. These two hypotheses are reconciled here, since the main burst of speciation in this group probably did take place in Argentina or Chile (*gymnocercus-fulvipes-*[*griseus+culpaeus*]). As suggested by Yahnke *et al.* (1996), *L. fulvipes* may represent a set of relict populations of a once more widely distributed species, whose phylogenetic affinities within this group are still not completely settled. Future work should use increased sampling of individuals and characters to attempt to clarify this issue so that a more complete biogeographic inference can be devised for this group. Moreover, the prospect of fully resolving this recent South American radiation promises to shed light on some of the processes shaping the composition of mammalian communities in this region during the Pleistocene.

## Supplementary Material

The following online material is available for this article:

Table S1 - Samples analyzed in the present study.

Figure S1 - Phylogenetic relationships among *Lycalopex* spp
