# Supplementary material for: Molecular assessment of the phylogeny and biogeography of a recently diversified endemic group of South American canids (Mammalia: Carnivora: Canidae)
Source: Genet Mol Biol. 2016 Jul 25;39(3):442–51. doi: 10.1590/1678-4685-GMB-2015-0189 (PMC5004827; doi:10.1590/1678-4685-GMB-2015-0189)
Supplement: Supplementary file 1 [file 1415-4757-gmb-1678-4685-GMB-2015-0189-Suppl01.pdf]

Table S1 - Samples analyzed in the present study. Collection site codes refer to localities shown in Figure 1. Haplotype codes are the same as shown in Figures 2-3.

Species	***Collection site code***	***Samples***	**Haplotype**	**Institution / Contact**
Lycalopex culpaeus	Junin de Los Andes - Neuquen Province – Argentina	ZCU6, ZCU31, ZCU45	C01	WCS / Andres Novaro
		ZCU57, ZCU59, ZCU 60, ZCU 65, ZCU70	C03	
		ZCU 41	C04	
		ZC 282	C05	
		ZCU24, ZCU29, ZCU39, ZCU51, ZCU52, ZCU53, ZCU68, ZCU69, ZCU71, ZCU72, ZCU73, ZCU74	C07	
		ZCU13, ZCU14	C08	
		ZCU 48	C09	
		ZCU46, ZCU47, ZCU56, ZCU75	C10	
	Central Chile	Dcu702	C11	LGD / Warren Johnson
	Unknown locality [1]	Dcu	C06	UCLA / Robert Wayne
		CVA05	C02	
Lycalopex fulvipes	Nahuelbuta Park - Chile	Dfu 01	F01	LGD / Warren Johnson
		Dfu 02	F02	
		Dfu03	F03	
	Ancud-Chiloé-Chile	CVAH001	F03	UCLA / Robert Wayne
	Peyuco beach – North of Valdivia-Chile	CVADAR, CVADARW	F03	UCLA / Robert Wayne
Lycalopex	Sierra de La Ventana–	AR31 [2]	GR08	Mauro Lucherini

griseus	Argentina			
	Central Bolivia	Pgy3 [2]	GR07	WCS / C. Fiorello
		Pgy4 [2]	GR06	
		Pgy5 [2]	GR04	
		Pgy6 [2]	GR05	
		Pgy7 [2]	GR09	
	Nahuelbuta Park - Chile	Dgr1, Dg6	GR16	LGD / Warren Johnson
		Dgr16	GR02	
	Conguillo Park - Chile	Dgr502	GR17	LGD / Warren Johnson
	Central Chile	Dgr711	GR17	LGD / Warren Johnson
		Dgr715	GR11	
	West of Purranque – Chile	CVAE01	GR17	UCLA / Robert Wayne
		CVAE02	GR15	
	Peyuco beach – North of Valdivia – Chile	CVAC02	GR12	
		CVAC01	GR03	
	Lago Rupanco-Chile	CVAA02, CVAA01	GR 12	
	Osorno-Chile	CVAG01	GR12	
	Chaihuin- Valdivia-Chile	CVAF001	GR14	
	Peyehue Park –Chile	CVAB01	GR12	
	unknown locality [1]	DGR4, DGR6	GR16	
		CVA02	GR13	
		DGR8	GR05	

		DGR12572	GR10	
		Dgr4W	GR12	
		DGR3	GR01	
Lycalopex gymnocercus	Taim – RS state - Brazil	bPgy30	G11	UFLA / Alex Bager
		bPgy32	G18	
		bPgy33	G14	
		bPgy35, bPgy41	G05	
		bPgy37	G02	
		bPgy38	G17	
		bPgy39	G14	
		bPgy 42	G15	
	Northern RS state – Brazil	bPgy06	G10	UFRGS / Tatiane Campos Trigo; Ana Paula Brandt
		bPgy09, bPgy18, bPgy19	G08	
		bPgy29	G06	
	Western RS state - Brazil	bPgy10	G03	
		bPgy13	G13	
	Eastern RS state - Brazil	bPgy07	G12	
		bPgy05, bPgy16	G07	
		bPgy17	G09	
	RS state – unknown locality [1]	bPgy01	G16	PUCRS / Eduardo Eizirik
		bPgy 02	G04	
		Dtg02	G13	

		Pgy	G01	
Lycalopex sechurae	Unknown locality	Dse	S01	Yahnke *et al.,* 1996
Lycalopex vetulus	Goias State - Brazil	bPve04	V12	Goiânia Zoo / Roberto Portela
		bPve05	V13	
		bPve 06	V16	
		bPve07	V11	
		bPve 10, bPve 11, bPve 12, bPve 14, bPve 15, bPve 17	V01	
	São Paulo State - Brazil	bPve 08, bPve 09	V10	Cristiana Prada; Denis Sana
		bPve01	V16	Instituto Pró-Carnívoros
	Maranhão State – Brazil	bPve 13	V02	Instituto Pró-Carnívoros
	Piaui State - Brazil	bPve 16	V02	Instituto Pró-Carnívoros
	Mato Grosso state - Brazil	bPve 18, bPve 21, bPve 22, bPve 24	V03	Instituto Pró-Carnívoros
		bPve 19	V05	
		bPve 20	V07	
		bPve 23	V06	
		bPve 25	V04	
	Minas Gerais state - Brazil	bPve03	V09	UFRGS / Ligia Tchaicka
	Bahia state - Brazil	bPve02	V08	UFRGS / Ligia Tchaicka
	Unknown locality [1]	Dve 12	V14	UCLA / Robert Wayne
		Dve 13	V15	

[1] Samples collected from captive animals; [2] Samples originally identified as *L. gymnocercus* by field researchers, and collected in areas where only this species is expected to occur
