# Supplementary material for: Molecular assessment of the phylogeny and biogeography of a recently diversified endemic group of South American canids (Mammalia: Carnivora: Canidae)
Source: Genet Mol Biol. 2016 Jul 25;39(3):442–51. doi: 10.1590/1678-4685-GMB-2015-0189 (PMC5004827; doi:10.1590/1678-4685-GMB-2015-0189)
Supplement: Supplementary file 2 [file 1415-4757-gmb-1678-4685-GMB-2015-0189-Suppl02.pdf]

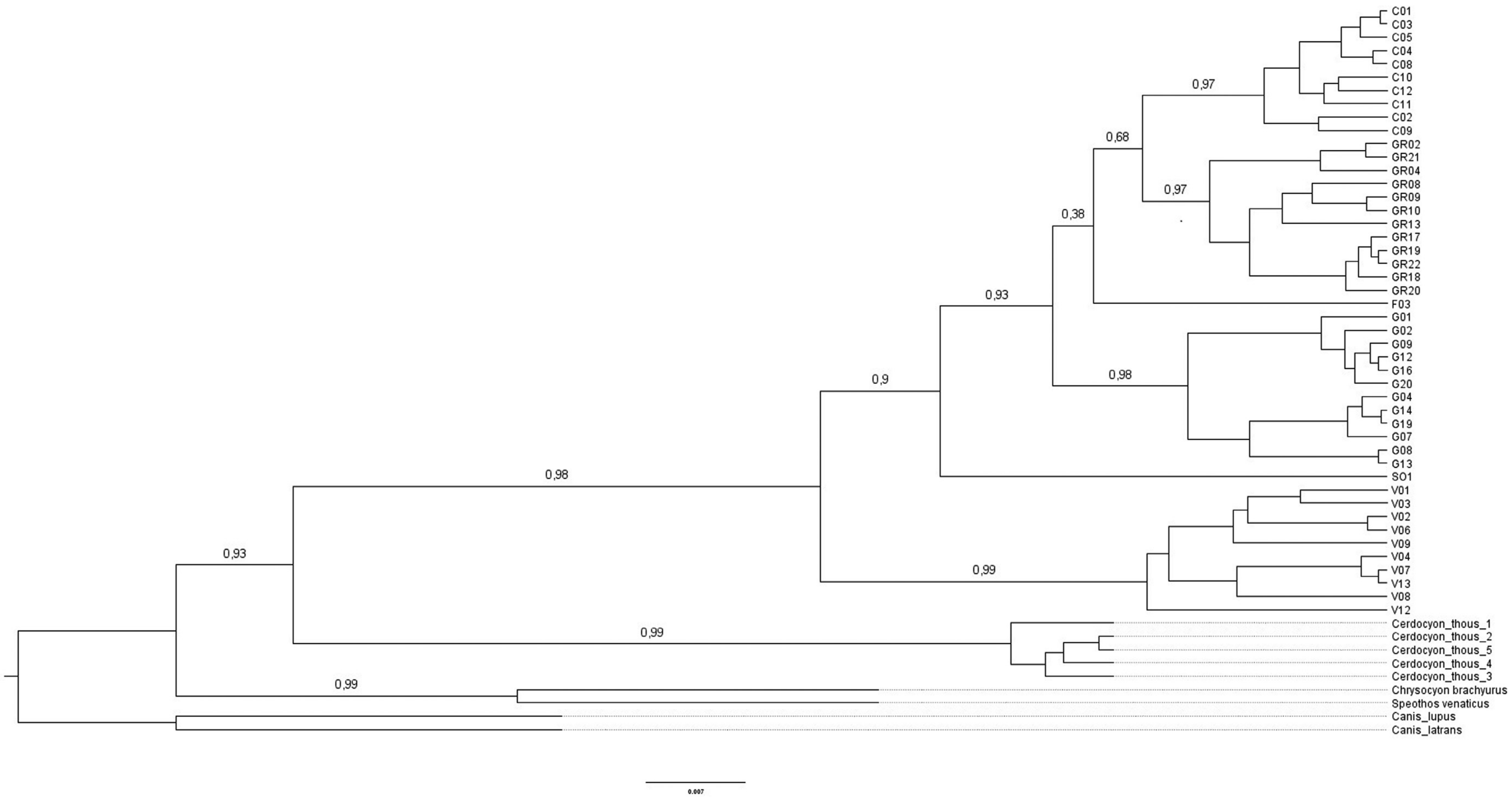

Figure S1 - Phylogenetic relationships among *Lycalopex* spp. inferred on the basis of mtDNA control region data. Analysis settings are the same as in Figure 2, with the inclusion of two additional outgroups (*Chrysocyon brachyurus* and *Speothos venaticus*).
